# Assessing the Feasibility and Acceptability of Health Coaching as a New Diabetes Management Approach for the People with Type 2 Diabetes in Saudi Arabia: A Protocol for a Mixed Methods Feasibility Study

**DOI:** 10.3390/ijerph192215089

**Published:** 2022-11-16

**Authors:** Abdullah N. Almulhim, Elizabeth Goyder, Samantha J. Caton

**Affiliations:** 1 School of Health and Related Research (ScHARR), University of Sheffield, Sheffield S1 4DA, UK; 2 Public Health Department, College of Health Sciences, Saudi Electronic University, Riyadh 13316, Saudi Arabia

**Keywords:** health behaviour change, health coaching, self-management, behaviour change techniques, type 2 diabetes

## Abstract

Background: Over recent years, the Middle East, and especially Saudi Arabia, has faced multiple changes, including structural-demographic and economic shifts. This has led to massive changes in the population’s lifestyle, including more unhealthy diets and increases in physical inactivity. As a result, accelerating rates of chronic diseases, including type 2 diabetes mellitus (T2DM) are a major public health concern. Current diabetes care in Saudi Arabia focuses on increasing the awareness of patients through various approaches, mainly based on health education, which is found to be suboptimal and ineffective for improving long-term outcomes. This study aims to assess the feasibility and acceptability of using a client-centred approach called health coaching that supports, enables, and engages T2DM patients to take the central role of controlling their own conditions by developing new crucial skills. Methods: A mixed methods randomised controlled feasibility study of health coaching will be used. Participants (n = 30) are adults with T2DM with poorly controlled diabetes (A1C ≥7) who can read and write in Arabic. Eligible participants are randomly allocated to either an intervention or control group for 12 weeks. COM-B model and Behaviour Change Technique Taxonomy version 1 (BCTTv1) guide the intervention curriculum. Predetermined progression criteria will be used to determine whether to proceed to a larger trial or not. Outcomes will be measured at baseline and 3 months. The study’s primary aim is to assess the process of eligibility, recruitment, retention and completion rates, acceptability and suitability of intervention and the time to complete each procedure. The preliminary efficacy of health coaching is the secondary outcome that includes different measurements, such as HbA1c, blood pressure, body mass index (BMI), waist circumference, weight, patients’ self-efficacy, and diabetes self-management. Discussion: This is the first study to explore the feasibility, acceptability, and preliminary efficacy of health coaching that used the Capability, Opportunity, Motivation, Behaviour (COM-B) model and BCTTv1 as guidance to develop the intervention for adults with T2DM in Saudi Arabia. The findings of this study will be used to inform the larger RCT trial if it is shown to be feasible and acceptable.

## 1. Background

Type 2 diabetes mellitus (T2DM) is a widespread chronic disease that poses a real worldwide health threat that is becoming a significant public health concern. T2DM is profoundly impacted by an excess body mass index (BMI) and physical inactivity [1]. Diabetes contributes to lead patients developing severe complications such as heart and stroke disorders, eye problems and complications with hearing, kidney failure, nerve injury, amputations, oral issues, and foot problem [2]. Adults with diabetes are two or three times more likely to develop heart problems and strokes [3]. In addition, there are other negative impacts of diabetes on social life, economic status, and health care services. Since diabetes is a preventable disease, which is strongly related to and affected by lifestyle, self-management is an optimal way to effectively manage it [4].

Worldwide, and especially in Saudi Arabia (SA), the prevalence of T2DM is growing at an alarming pace. According to a WHO report (2017), SA has the second highest rate of T2DM and is ranked the seventh highest in the globe. Additionally, the prevalence rate of T2DM has increased tenfold in the past three decades in SA. About 89% to 97% of all Saudi patients with diabetes are diagnosed with T2DM [5]. About seven million people suffer from T2DM in Saudi Arabia and three million are pre-diabetic. T2DM affects approximately 30% of the Saudi population and this increases with age [6]. The prevalence of T2DM is higher among women than men and with higher BMI individuals [6]. Moreover, it is more than likely that the burden of this chronic disease will only increase owing to factors such as an increased rate of obesity, an ageing population, changing lifestyles and poor diet [7]. The widespread increase in sedentary behaviour and physical inactivity among Saudis contributed to increasing the prevalence of T2DM [8] In SA the high rate of T2DM is associated with the high prevalence of obesity as a result of the fast urbanisation that led to adopting modern western dietary habits such as fast-food and increasing the level of physical inactivity [9].

The healthcare system in SA is still trying to address the alarming challenge of T2DM through several national programs and initiatives, but the outcome is still suboptimal. In SA, several studies have provided evidence that T2DM is not managed effectively [10]. While the current attempts are heavily reliant on awareness-raising campaigns, T2DM patients are still struggling to adopt a healthy diet and lifestyle to manage their conditions effectively. Adopting a healthy diet and lifestyle is an essential element of effective self-management of diabetes [11]. It is noteworthy that lifestyle interventions seem to be at least as effective as medications [12].

Diabetes education-based programs have not always been entirely adequate in bringing about the desired change. Many studies make it clear that awareness alone does not contribute to better self-management practices [13]. The responsibility of controlling T2DM is largely dependent on individual behaviour. T2DM patients are the key players to address this issue, so they need enough time to address their needs to improve their self-management skills. Healthcare providers often do not have adequate time for a face-to-face discussion with patients since regular visits at primary care clinics are usually restricted to 10 to 15 min, which leads to patients feeling negative, overwhelmed, and frustrated. Cost-effective interventions are needed to bridge the gap by listening to patients and helping them to overcome potential barriers. Developing a patient’s self-management skills is crucial to delay or reducing the risk of T2DM. The occurrence of the chronic conditions associated with T2DM can be remarkably diminished or delayed—by 75% or more—with the help of healthier lifestyle behaviour choices made by patients [14]. Self-management interventions are needed that enable patients to control and manage T2DM [15] consistent with the current diabetes treatment philosophy that promotes patient-centred approaches [16].

There have been recent attempts to improve diabetes care in SA with T2DM through diabetes self-management programs. These programs are very uncommon and not gaining widespread adoption [17]. Most lifestyle interventions still rely on education as the core element to improve patients’ self-care. The impact of diabetes education programs is suboptimal [17]. However, most studies have emphasised the significant need to shift the current attention toward employing new approaches that help patients to develop self-management skills. A recent systematic review, conducted on gulf cooperation council countries, included studies in T2DM self-management between 1996 and 2015 and found that none of the studies employed the skills needed to improve patients’ self-management capability, such as problem-solving skills [5]. Moreover, none of the studies has employed a patient-centred approach to work with a patient as an ally rather than an educator. Client-centred interventions that are tailored to the patient’s needs are more likely to bring about desired outcomes [18].

Health coaching is a client-centred approach that is derived from different disciplines and behaviour change theories. It has become an emerging trend in the literature and is gaining widespread acceptance in the health promotion fields but this one-on-one type of tailored intervention has not yet been used in Saudi healthcare systems. Different studies have revealed that health coaching as an intervention centred on patients’ values may provide added benefits to traditional T2DM education programmes [19]. It can be a highly effective and financially viable model when it focuses on improving patients’ self-efficacy and important skills, including goal-setting, and problem-solving [20]. Health coaching has been applied in multiple different contexts and showed as an effective tool to improve diabetes self-management [21,22].

However, recent systemic reviews of randomised controlled trials utilising health coaching have reported mixed results, with some reporting that health coaching is effective, while others claim it is ineffective [21,22]. One of the explanations for the inconsistent reported findings across different studies is the lack of agreement on the active ingredients and content of effective health coaching interventions.

There is currently no consensus in the literature on designing an effective health coaching intervention, including the selection of a suitable theoretical basis and active ingredients, or behaviour change techniques [22]. In the absence of such consensus, there is uncertainty towards which coaching methods are more appropriate and effective to use including intervention content as well as the duration, length, and mode of delivery of sessions, particularly when applying health coaching in a new setting [23]. A lack of guidance, ineffective methods used to develop interventions, inappropriately selected intervention components and variation in the reporting of outcomes have been suggested to contribute to the mixed effectiveness of health coaching interventions [24,25,26]. Furthermore, health coaching interventions that have been developed and have succeeded in Western or other contexts may not be effective for people in different contexts such as SA. Consequently, such interventions may require adaptation and modification to make them more feasible, acceptable, and suitable in the context of Saudi society.

A feasibility study is highly recommended prior to a full-scale trial to test potential uncertainties, especially when a complex intervention is to be applied in a new context [27]. Feasibility studies help to explore specific issues such as study protocol, context-specific relevance, practicality, and the possibility of efficacy [27,28]. In addition, feasibility studies help examine the procedures’ acceptability, assess the recruitment and retention process, and determine the appropriate sample size for a future full-scale trial [27].

To the best of our knowledge, none of the previous health coaching programs have used the BCW model and BCTs taxonomy as a guide to build the intervention content systematically in order to analyse and achieve planned outcomes. This is aside from the fact the application of diabetes self-management studies within the Saudi context is very uncommon and often focuses on providing health education [17]. Moreover, none of the self-management interventions have engaged stakeholders during the intervention development process, so the interventions may have failed to bring about the change and address the difficulties patients encounter in managing their diabetes [17].

As a result, the future full-scale intervention would be effectively refined and/or inactive ingredients replaced with other appropriate BCTs. Careful selection of theoretical framework and BCTs prior to the intervention being conducted is crucial and widely recommended [29].

### 1.1. Study Aims

The present feasibility study aims to determine whether the health coaching intervention for type 2 diabetes is feasible and acceptable to improve self-management and reduce HbA1C to inform a full-scale RCT trial in SA. The objectives of the study are categorised into the process evaluation objectives (primary) and outcome (secondary) objectives. The process evaluation objectives are related to the implementation phase and mainly focus on the feasibility of processes and procedures of the study. The outcome objectives are related to the efficacy of the intervention.

#### 1.1.1. Primary Objective: (Study Feasibility-Process Evaluation-Objectives)

To assess the feasibility of conducting a future definitive study to evaluate the effectiveness of using an adapted health coaching intervention in the Saudi context by:

1. Assessing recruitment, and retention rates, and estimating the effect size;
2. Assessing the implementation process, including data collection procedures;
3. Assessing and determining the sample size for the larger-scale trial using the findings of this feasibility study;
4. Exploring the acceptability and suitability of intervention through participants’ perceptions of, and experiences with, the health coaching intervention.

#### 1.1.2. Secondary Objective: (Outcome Objectives)

To assess the preliminary efficacy of health coaching to improve diabetes self-management and reduce HbA1C by evaluating its influence on participants’ behaviours and their self-management ability compared to usual diabetes care by:

HbA1c and other variables will be assessed pre- and post-intervention at baseline and at 3 months. The intervention’s clinical goal for T2DM self-management is an HbA1c level of <7.0%. Other variables will also be assessed; namely, glycosylated haemoglobin (HbA1C), blood pressure, body mass index (BMI), waist circumference, weight, patients’ self-efficacy, and diabetes self-management.

## 2. Methods

The protocol of this study is following the guidance of CONSORT [30]. The methods are categorised into subsections presented below (see Supplementary File S1).

### 2.1. Study Location

Saudi Arabia will be the location of this study. The trial will only be conducted at one site, namely the obesity, endocrine, and metabolism centre at King Fahad Medical City (KFMC), the hub of the Riyadh Second Health Cluster. KFMC is one of Saudi Arabia’s most significant and well-known medical cities, which serves the country’s largest medical complex.

### 2.2. Trial Design

The present study will adopt a double-blind randomised two-arm feasibility trial to investigate the feasibility of a 3-month intervention for participants who have had difficulty managing their T2DM. Mixed methods will be employed in the study’s design to gather and analyse both quantitative and qualitative data. A mixed methods approach allows the study to address additional research questions [31]. An RCT will be used since it is the best design for comparing the effectiveness of health coaching between the two groups [32]. The primary and secondary trial outcomes will be examined using various approaches, including questionnaires, interviews, focus groups, and clinical measures, to assess the intervention’s feasibility, acceptability, and preliminary effects. The study evaluation will be conducted at two points: at the intervention’s baseline and three months following the intervention (endpoint). To conduct this study, ethical approval was obtained by the University of Sheffield and the Institutional Review Board (IRB) committee form ethical approval at (KFMC) (IRB Log Number: 21-062E). The CONSORT flow diagram will describe the route participants take through the health coaching intervention, including the schedule of enrolment, interventions, and assessments [33].

### 2.3. Participants

The target population of this study is adult men with T2DM (HbA1c) ≥7.0%. Participants will be eligible for recruitment as per the following criteria:

### 2.4. Eligibility Criteria

- Aged >18 years old;
- The participant diagnosed with T2DM;
- Haemoglobin A1c (A1c) ≥7.0%;
- The participant can read and understand Arabic;
- The participant has access to a personal mobile phone/smartphone;
- The participant is willing to complete the intervention period;
- The participant is willing to remain in Riyadh.

Patients are not eligible if they: (1) had physical impairments that prevented them from participating in physical activity, (2) patients are unable to understand or unwilling to give their informed consent.

### 2.5. Recruitment

Recruitment will take place at a primary care centre at KFMC. Advertising for the intervention will use multiple methods, including posters, brushers, social media and through health care provider referrals. Interested individuals will be assessed for eligibility to participate in the intervention. Those who meet the eligibility criteria will be contacted to meet with the research team to briefly explain the study and answer any related questions. Interested individuals will be given a booklet that explained the study and its aims before signing the consent form. Participants will complete a short initial screening assessment, including demographic information and baseline-related laboratory reports.

### 2.6. Randomisation and Blinding

All recruited participants will be randomised on a 1:1 ratio into one of two groups. The randomisation process will be carried out by an independent person out of this study and used a computer to generate random numbers. To minimise selection bias, eligible participants will have an equal opportunity of being allocated to each group. The researchers will remain unaware of which group a participant had been assigned to before the baseline assessment. Participants will be informed which group they have been assigned to after the baseline assessment.

### 2.7. Sample Size

To the best of our knowledge, there was no previous health coaching RCT feasibility study in the field to use as a reference for the sample size. Thus, this study seeks to recruit at least 9% (n = 30) of the sample size required to carry out a full trial [34], with a minimum of 12 participants in each group [35]. Participants will be randomly allocated to either the coaching group (n = 15) or the control group (n = 15).

### 2.8. Intervention

#### 2.8.1. Intervention Content (Intervention Group)

Health coaching in the context of T2DM can be characterised as a complex intervention due to the various direct and indirect interacting components that impact the intervention’s outcomes. Health coaching, as a complex approach, has arisen from multiple different behaviour change theoretical bases. While complex intervention is not a straightforward study with direct clear causal and effects interaction, several crucial requirements and careful considerations are required when replicating it. This is aside from the fact that health coaching combines various disciplines such as psychology with numerous theories into one approach. Thus, we used the model and Theoretical Domains Framework (TDF) as guidance in adapting the intervention into a new context, namely Saudi Arabia.

The intervention is drawn on the COM-B model and TDF to provide a precise and systematic description of the health coaching content. COM-B model is the central part of the Behaviour Change Wheel (BCW) that helps in identifying the interactive process from; capability, opportunity, and motivation required to bring about the desired behaviours of an intervention [23]. The Theoretical Domains Framework (TDF) is a tool to provide further comprehensive explanations that link the COM-B model for a better understanding of the needed changes and determining the target behaviour [36]. Several barriers have been identified based on a literature review of previous work and what needs to be changed to bring about the desired outcomes. In addition, different target behaviours have been identified that would lead to adopting a healthy diet and lifestyle as advocated by UK lifestyle guidelines [37]. To the best of our knowledge, there are no evidence-based guidelines in SA for T2DM to adopt a healthy diet and lifestyle. Therefore, the UK lifestyle guidelines will be used due to their relevance to the Saudi context [5,37]. The UK lifestyle guideline will be utilised to identify the possible behaviour targets. In order to specify target behaviours, a discussion will be conducted with health practitioners at the health centre (the intervention setting), including a dietician, to reach an agreement on the behaviours list. The discussion helps assess and prioritise the target behaviours according to their potential effects, and possibility to be measured and achieved [38].

The four proposed behaviour targets are [37]:

- Decrease carbohydrate intake for each meal;
- Use unsaturated fats as much as possible (avoid saturated fats);
- Do exercise for 30 min, five days in a week;
- Monitor waist circumference, maintain it below (80 cm for women and 94 cm for men).

Based on the results from COM-B and TDF behaviour analyses, different intervention functions will be identified and linked to the key barriers (see Table 1). Next, the intervention functions will be assessed for their suitability based on APEASE criteria; affordability, practicability, effectiveness/cost-effectiveness, acceptability, safety/side effects and equity, as recommended by the BCW guide [23].

As recommended by BCW guide, multiple BCTs could be used for each identified intervention function [23]. The BCW guide suggests the most common and less frequently used BCTs for each intervention function. Different possible BCTs were identified and included as a part of intervention functions to deliver certain coaching activities and address the key barriers. The BCW guide, previous literature review analysis, including recent systematic review results for the most effective BCTs associated with a clinically significant reduction in HbA1C were all used to help in selecting BCTs. Some BCTs were selected as per the competencies of health coaching as recommended by ICF and the intervention-underpinning theories. As advocated by [39], the BCTs have been selected and matched to the underpinning theories used in this study motivational interviewing (MI) and the transtheoretical model (TTM). The BCTs used that match the MI techniques are presented (see Supplementary File S2) [40]. In addition, other BCTs used in the health coaching intervention that match the TTM are presented (see Supplementary File S3) [41]. All the identified BCTs were assessed according to APEASE criteria to ensure the possibility of using them in the intervention context. Table 2 maps the BCTs selected and their link to the key barriers and intervention functions.

In summary, a total of 29 BCTs will be used to carry out the content of health coaching, including the activities to achieve the desired outcomes. Out of 29 BCTs, 16 BCTs were identified from the intervention underpinning theories (IM and TTM) [40,41]. In addition, four BCTs were identified and showed effectiveness in reducing the levels of HbA1c [42]. Additional nine BCTs were included that match the provided skills of health coaching.

#### 2.8.2. Intervention Procedure

**Figure 1:**
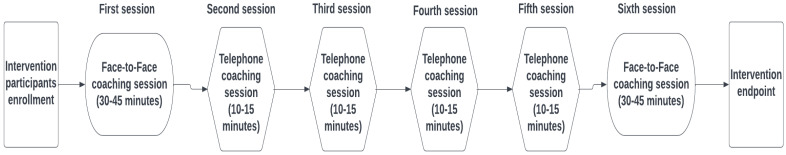
Health coaching intervention sessions biweekly.

Three trained and qualified health coaches will deliver the coaching sessions. They all will receive training on the intervention curriculum (see Table 2). Additionally, they will be monitored by the researchers throughout the period of delivery of the intervention. The intervention consists of 6 sessions that will be delivered biweekly via face-to-face meetings and telephone coaching over a 3-month period. Intervention group participants will be contacted fortnightly and coached using a combination of different methods; in person, by phone, and by smartphone (e.g., secure messaging such as WhatsApp) while the control group participants only receive the usual care for participants with T2DM with includes general lifestyle advice. Face-to-face coaching is only conducted at baseline and in the third month of the intervention for 30–45 min coaching sessions. In addition, 10–15 min telephone coaching will be delivered for the other four sessions (see Figure 1). 

The health coach works as an ally to enable participants to effectively engage in their self-care needs and address potential self-management barriers. They will be provided with different tools, including a patients’ progress tracker, coach notes log and a coaching log to help them run each coaching session. Participants will be given the opportunity to learn and practice significant skills to help them reach target goals through action plans they set for themselves for better self-care [43]. Table 2 illustrates the operationalised protocol and phases of session delivery

#### 2.8.3. Usual Care (Control Group)

Control group participants will receive only the usual care. Standard care includes providing written information on diabetes and brochures for raising awareness, including the benefits of modifying their health behaviours. Generally, T2DM patients have regularly scheduled visits to check diabetes management by endocrine specialists. The visit’s main purpose is to check whether the patient’s medications need to be replaced, increased, or continue with the same prescription.

### 2.9. Measures

Different tools and measures will be used to assess the primary and secondary study’s outcomes as presented in below sections. To reduce the risk of bias, an independent interviewer will be trained and supervised by the researchers to undertake the role of collecting some data, including semi-structured interviews and providing questionnaires. The interview guide will be developed by the researchers and used by the interviewer to guide the interviews. The interviewer will remain blind to the intervention details, process, and activities. Control group participants will be requested to complete pre- and post-intervention measures and questionnaires.

#### 2.9.1. Primary Outcome Measures

##### Feasibility

The primary outcome will assess the feasibility, including recruitment rates of the study and retention, and acceptability of the intervention by participants. During this step, the researchers seek to measure the possibility of recruiting sufficient participants within a specific period and the number of recruited participants who completed the study. Participants’ responses and expressions of their engagement interests will be recorded, including reasons for interest, reasons for engagement, and reasons for not interest. The number and percentage of interested eligible, complete, and drop-out participants will be recorded to explain the recruitment rates and retention. In addition, the researchers will undertake a qualitative process evaluation to investigate participants’ and their physicians’ experiences and feedback on the intervention, including assessment of the most appropriate advertising method for the intervention. Different questionnaires will be used to provide further information. In addition, the health coach also will report on his experience with participants.

##### Acceptability

Qualitative and quantitative data will be used to assess to what extent the intervention and its implementation are acceptable to the patients. The purpose of assessing the implementation process is to identify any potential problem with the methodology used to deliver the intervention. The acceptability of the implementation process, including data collection procedures, will be assessed by conducting post-intervention semi-structured qualitative interviews, informal meetings and focus groups. Interview guidance will be used to direct the conversations and enable the researchers to explore more details about how the participants find and describe their intervention experience. A questionnaire will be used to quantify the participants’ self-reported satisfaction (Likert-scale Satisfaction Questionnaire, 14-items). Healthcare providers and participants’ opinions are important to help adapt the intervention in terms of ensuring that it corresponds with and is acceptable for the Saudi cultural context.

#### 2.9.2. Secondary Outcome Measures

The secondary outcomes (preliminary efficacy) comprise anthropometric, clinical, and psychological variables selected as of the standard measures for monitoring and prevention of complications in diabetes [44]. These variables will be assessed twice at the intervention, at baseline and after 3 months for HbA1C, blood pressure, body mass index (BMI), weight and waist circumference. Change in outcomes post-intervention will help to determine if the intervention is feasible, acceptable, and effective. Table 3 provides the timeline for the assessment of the variables.

#### 2.9.3. Demographic Information

Demographic information will be used to explain the nature of the recruited participants in the intervention. It will be collected at a single time during the intervention baseline.

#### 2.9.4. Fidelity Assessment

Qualitative data, including interviews, focus groups, meetings, and coaching sessions will be audio recorded, transcribed, and translated into English. Thematic analysis will be used to analyse qualitative data. Multiple audio recorders will be used to ensure high reliability, and they will be transcript to text by two independent native Arabic speakers. Final coding will be used after reaching an agreement between the independent transcribers. Translation to English will be the next step completed by the researchers and an additional check of meaning by an independent native Saudi Arabian Arabic speaker.

#### 2.9.5. Predetermined Progression Criteria to Proceed to a Larger Trial

Before the feasibility of RCT started, the research team developed progression criteria. The progression criteria will help to decide on carrying out a full scale based on the findings generated from the feasibility study regarding recruitment, retention, adherence, and overall acceptability [45]. These criteria establish the benchmarks for assessing the trial’s feasibility and determining whether undertake a further larger-scale study. The predetermined progression criteria are presented in Table 4.

#### 2.9.6. Data Management

Both textual and audio data will be generated and collected using different research methods. The textual data are (i) clinical observations and field notes; (ii) notes collected from formal and informal meetings with healthcare staff; and (iii) field notes from the health coaching sessions. The audio data will consist of (i) recordings of some formal and informal meetings with healthcare professionals; (ii) recordings from health coaching sessions; and (iii) recordings from focus groups. All recordings will be immediately downloaded on separate devices.

The majority of raw quantitative data will be stored as tabular data copies in Microsoft Excel format to allow for statistical analysis via (SPSS). Thematic analysis will be used to analyse qualitative data. Multiple audio recorders will be used to ensure high reliability, and they will be transcript to text by independent native Saudi speakers. Qualitative data will be stored temporarily on multiple hard drives, e.g., USB and Google Drive, and manual copies, e.g., handbooks and logbooks. Separate files will be used for each type of qualitative data for organising purposes. For ensuring the long-term usability of data, they will be stored in a secure place within (KFMC governmental institution) for confidentiality purposes and participants’ privacy. Additional space storage will be requested from the University of Sheffield to ensure that enough multiple copies of data are there in case of any potential risk, for example, losing some data due to storage abilities or difficulty in accessing any other technical issues. I will continuously make backups of the files on external hard devices and in a safe and secure place through (KFMC and the University of Sheffield).

Qualitative data, including interviews, focus groups, meetings, and coaching sessions will be recorded on electronic recorders, transcribed, and translated. There will be interview guidance used to direct the conversations and enable the researchers to obtain the intervention’s information. All participants will be given a unique reference number used when referring directly to their responses. Quantitative data will be generated and collected by the researchers with support from the centre’s health professionals, including HbA1c, BMI, blood pressure, weight and waist circumference. All data will be encrypted using a high-level encryption method to protect collected data (using FileVault on Mac) and the University Data Centre.

The researchers will be monitoring the quality of data from the beginning until the intervention endpoint. Data will be organised based on its type (e.g., interviews, coaching sessions, etc.), in separate files within one folder for easy access to each type at any time. Multiple copies of the folder will be backed up and stored throughout the period of data collection. Backups with up-to-date data will be completed continuously to keep data safe and updated. Qualitative data will be recorded to avoid accidentally missing information. This would allow for assessing the consistency and quality of the data collected. Vitro measures will be taken by a health practitioner (e.g., laboratory specialist) from KFMC in Riyadh. Multiple samples will be taken to ensure the quality of results by comparison.

## 3. Data Analysis

### 3.1. Quantitative Data

The feasibility and acceptability of the study’s findings will be evaluated in relation to the quantitative data using descriptive analysis. The descriptive analysis covers different aspects as the following:

- Screening, recruitment process, retention and adherence (coaching sessions) rates will be calculated and presented as proportions
- Length of time to recruit the target sample
- Duration of time needed to complete the assessments
- Percentage of completed interventions sessions
- Average time needed to complete each session
- Description of participants interaction during coaching sessions (frequent BCTs used, interactions with coaches)
- Acceptability and suitability of intervention through participants’ perceptions of, and experiences with, the health coaching intervention (Satisfaction Questionnaire)
- Additionally, other outcome measures such as diabetes self-management and patient self-efficacy will be evaluated at baseline and endpoint to investigate changes in participants’ behaviours compared to another group
- Preliminary effects of the intervention
- Regarding the evaluation of the preliminary efficacy of the intervention, secondary variables will be described by means and S.Ds, investigated pre- and post-intervention to measure changes in the outcomes; glycosylated haemoglobin (HbA1C), blood pressure, body mass index (BMI), waist circumference, weight, patients’ self-efficacy, and diabetes self-management
- Correlations will be performed between:
- The number of completed coaching sessions and A1c, BP, BMI, WC body weight at 3 months
- The number of completed coaching sessions and self-efficacy and diabetes self-management scales at 3 months

### 3.2. Qualitative Data

Qualitative data, including interviews, focus groups, meetings, and coaching sessions will be audio recorded then transcribed, and translated into English. Thematic analysis will be used to analyse qualitative data. Multiple audio recorders will be used to ensure high reliability, and they will be transcript to text by independent native Saudi Arabian speakers. The qualitative analysis includes the following:

All interviews and focus groups are recorded and transcribed

- A total of 10% of transcription will be translated from Arabic to English, back-translated for accuracy and validity purposes, and checked by a professional native speaker.

To prevent meaning from being lost in translation, to improve the validity of the data, which might be compromised if it is translated, and to expedite the process, the analysis will be carried out in the original language [46].

- The analysis will be conducted by using:
  o A reflexive thematic analysis [47];
  o From a Pragmatic philosophical standpoint [48];
  o Thematic analysis will be manually conducted using NVivo software.

Next, all interactions with the health coaches through the coaching sessions will be mapped to the BCW and the BCTTv1 using NVivo for coding used BCTs. The final step is integrating QUALI and QUANT data and analysing them as they occurred concurrently simultaneously per the thesis process diagram (convergent design).

## 4. Discussion

Changing lifestyle is widely recognised as the key to supporting people with T2DM to adopt healthy behaviours to control their condition. An individualised, self-management approach for non-communicable diseases such as T2DM is now urged to a more prominent degree than at any other time [49,50]. Several studies have reported the positive effects of lifestyle intervention programmes among people at high-risk, with a 58% decrease in the incidence of T2DM [51,52]. Utilising a lifestyle intervention aims at reducing the probable risks of chronic diseases in addition to decreasing the occurrence of such conditions, if already existent, as part of a management plan. Adopting a healthy diet accompanied by physical activity is likely to decrease the risk of T2DM by 45% regardless of genetic risk [11]. Different studies have found that chronic diseases such as T2DM can be effectively addressed using health coaching programmes, which aim to promote healthy behaviours [53]. Moreover, studies have demonstrated the effectiveness of health coaching with a variety of chronic illnesses, including T2DM [21]. 

Conducting the feasibility study will help inform the research development process by identifying key uncertainties in the study before a future full-scale intervention is carried out. It is hoped that the findings of the present study will contribute to informing the research field of health coaching on T2DM and to ensure the intervention achieves a good fit with a new context; namely, Saudi patients with T2DM. Since health coaching as a new approach is now included in the Saudi Ministry of Health’s (MOH) future plans, the study findings can directly inform future policy developments in SA. Additionally, it is hoped that the present study will make a significant contribution to the literature as it represents the first study that has been developed based on the use of specific “active ingredients” (BCTs), which directly match the intervention’s underpinning theories. Consequently, health coaching intervention designers may benefit from the study’s findings by allowing them to interpret and identify effective BCTs to facilitate future replication. Finally, the study’s findings could lead to the increased uptake of other behaviour change interventions in the Saudi Arabian context.

## Figures and Tables

**Table 1:** The BCTs selected and its link to the key barriers and intervention functions.

COM-B		TDF	Barrier	Intervention Function	BCTs
Capability	Psychological	Knowledge	Poor T2DM knowledge affect self-control Poor nutrition knowledge (what the association between diabetes and diet, type of healthy food) Poor knowledge about PA (how to engage in PA, how much time spend on PA, PA intensity, PA types, underestimate the role of PA	Education	5.1 Information about health consequences
	Physical	Physical skills	Lack of energy to do PA Lack or limited skills of physical skills to do exercise	Enablement Training	Enablement: 6.1 Demonstration of the behaviour 1.5 Review behaviour goal (s) 1.7 Review outcome goal (s) 2.3 Self-monitoring of behaviour 12.1 Restructuring the physical environment 4.1 Instruction on how to perform a behaviour’ 3.1 Social support (unspecified) Training: 8.1 Behavioural practice/rehearsal 8.3 Habit Formation 8.4 Habit reversal 8.7 Graded tasks 15.4 Self-talk 10.9 Self-reward 2.2 Feedback on behaviour 2.3 Self-monitoring of behaviour
Opportunity	Social	Social influences	Social norms and habits: overuse of high calories food intake; carbohydrates and fats, eating together (collectives), and social courtesy to eat unhealthy diet	Enablement	Enablement: 1.3 Goal setting (outcome) 1.1 Goal setting (behaviour) 3.1 Social support (unspecified) 1.4 Action planning 1.2 Problem-solving 2.3 Self-monitoring of behaviour 8.7 Graded tasks 5.5 Anticipated regret 12.1 Restructuring the physical environment 12.2 Restructuring the social environment
	Physical	Environmental context and resources	Lack of time Lack of resources (environmental, appropriate climate and financial ability) Lack of access to do activity Overuse of cars for transportations	Restriction Environmental restructuring Enablement Modelling	Enablement: 1.3 Goal setting (outcome) 1.1 Goal setting (Behaviour) 3.1 Social support (unspecified) 1.4 Action planning 1.2 Problem-solving 2.3 Self-monitoring of behaviour 9.2 Pros and cons 9.3 Comparative imagining of future outcomes 8.7 Graded tasks 1.9 Commitment 13.2 Framing/reframing 5.5 Anticipated regret 12.5 Adding objects to the environment Modelling: 6.1 Demonstration of the behaviour Environmental restructuring: 12.1 Restructuring the physical environment 7.1 Prompts/Cues Restriction: Use rules to reduce opportunity to engage in unwanted behaviour
Motivation	Reflective	Beliefs about own capability	Lack of willpower and self-confidence to do PA and maintain healthy diet	Persuasion Education Enablement	Persuasion: 15.1 Verbal persuasion about capability 15.2 Mental rehearsal of successful performance 9.1 Credible source 2.2 Feedback on behaviour 13.2 Framing/reframing 15.3 Focus on past success Education: 5.1 Information about health consequences 5.3 Information about social and environmental consequences Enablement: 1.9 Commitment 5.5 Anticipated regret
		Beliefsconsequences	Fear from consequences of PA (fear of injury and disease future complications)	Education	5.1 Information about health consequences
		Social role and identity	Struggle to change social identity associated with culture diet Struggle to accept the fact of living with diabetes	Education Persuasion	Education: 5.1 Information about health consequences Persuasion: 13.5 Identity associated with changed behaviour

**Table 2:** Health coaching intervention detailed protocol.

Phase #	Session Content	Session Goals	Intervention Function
1	Session #1, the patient’s assessment form and consent supposed to be completed General introduction about the health coaching intervention Outline the intervention structure and content Discuss the coach’s roles and the expectations from the participant (being completely clear with the client about the health coaching) Creating an alliance (Establish Trust) Learn from a patient (disease history, obstacles, priorities, strengths, goals, etc.) Help patient to create wellness vision Assess the readiness of patient’s stage in relation to change health behaviour (the transtheoretical model) Introduce the importance of having a healthy diet Introduce the importance of increasing physical activity Increase awareness of adopting a healthy lifestyle in relation to controlling diabetes Identify 3-month general behavioural goals and biweekly goals Explore resources needed to help achieve desirable behaviour Commitment	Identifying the patient’s current position in the overall health status (via the transtheoretical model) Build up a relationship (as an ally) between patient and coach Encourage open discussion Make sure the patient understands what health coaching is Allow the patient to develop a foundational conception of goal setting and action planning The patient creates (SMART) measurable, action-based, realistic and timely achievable goals Have better control of carbohydrate and fat intake Gradually increase physical activity Gradually increase achievable tasks until the intended behaviour is achieved	Education Enablement Training Restriction Environmental restructuring
2	Phase #2 (session # 2), (this phase will be used again in sessions # 4 and 5) Check ongoing progress Understand patient’s state (use reflections) Ask patient to share views (so far) of good things that occurred and experiences from last session Use positive reflections about patient’s strengths, passion or emotions Ask patient to assess the previously selected short goals and accomplishments Use reflections to understand potential barriers prevent patient from achieving past goals Identify specific strategies that they may use to overcome the obstacles Explore what patient learned from past experience Ask and discuss with patient next short goals Share feedback on patient’s progression Ask patient to connect current accomplishments to the general 3-months goals Review the general goals to see if patient want to revise them (to be more realistic and achievable) Affirm the patient’s choices, strengths, and capability Use techniques such as reflective listening to address ambivalence and respond to the patient’s resistance (motivational interviewing)	Assessment of progression Review goal setting (behaviour) Review behaviour goals to examine a patient’s performance progression toward the agreed goals Enable patient to develop problem-solving skills Enable patient to create action plan Prompt the participant to generate ideas and strategies to overcome barriers (problem-solving) Allow patients to monitor their behaviours (know the changes so far) Keep patient motivated (no matter the accomplishments)	Persuasion Education Enablement Training Restriction Environmental restructuring
3	Middle phase (session # 3), the coach continues to observe the patient and give feedback to help them move forward in achieving their goals through bi-weekly SMART goal setting. Patient continues to identify strategies to address existing obstacles and enhance their self-ability If goals are not achieved, the barriers will be identified, action plan will be taken to address these obstacles and modified goals will be created The coach will assess the patient’s self-efficacy by scoring goals to measure the participant’s confidence in achieving their goals The coach continues using the skills needed to explore ambivalence and discrepancies between the participant’s plans and their actual behaviour (Rollnick et al., 2005) Affirmations and appreciative inquiry will be used to appreciate progression and improve patients’ self-confidence	Assessment of current behavioural change Review goal setting (behaviour) Review behaviour goals to examine a patient’s performance progression toward the agreed goals Review all previous goals and reassess goal progress Enable the participant to assess their progress Prompt the participant to analyse factors influencing their behaviour Participant’s commitment to affirm to review and change behaviour	Persuasion Education Enablement Training
4	(session # 6) Conclude the coaching relationship Determine where the patient is in terms of their goals How the coach can best guide the client, and whether coaching is what will best benefit the client Participants’ assessment of the intervention, general satisfaction of the participant with the process Appreciate the patient’s engagement in the intervention Explore the patients’ experience and how future coaching intervention would best support T2DM	Allow patient to explore the difference at the endpoint Learn for participants’ experience Findings from the feasibility study will be used to justify an expansion of the study (full-scale) or refined for better outcomes so that we can carry out a large RCT on the efficacy of this intervention	Persuasion Education

**Table 3:** Timeline for the assessment of the intervention measurements.

Study Timeline				
Pre-Study Allocation			Post-Study Allocation	
Activity	Enrolment	Allocation	Baseline	Endpoint
Intervention advertising				
Screening eligibility				
Informed consent				
Baseline measures				
Randomisation				
Allocation				
Start of the Study:				
Intervention group				
Control group				
Assessments:				
Demographic				
BMI				
Weight				
Blood pressure				
Waist circumference				
HbA1c				
Feasibility Questionnaire				
Summary of Diabetes Self Care Activity (SDSCA), 12-items				
Self-efficacy Scale for Diabetes, 8-items				
Acceptibity Questionnaire				
Likert Scale Satisfaction Questionnaire, 14-items				

**Table 4:** Health coaching predetermined criteria.

Criteria	Predetermined Cut-Offs
Screening prospective participants	If 60–80% or more of those eligible to participate in the study consented, this supports conducting a large RCT scale trial, but if percentage is less than 50%, there is no significance to move forward.
Recruitment rate	If the recruitment rate of people who were eligible and consented was ≥80% this would support conducting a large RCT trial. If the rate was between 70–65%, this needs further discussion to explore the reasons and whether, if they can be modified, then the trial may progress with cautions. If the rate was ≤65%, there is no significance to moving forward.
Retention rate at 3-months	If the rate was ≥83%, this supports conducting a large RCT scale trial If the rate was less than 83%, there is no significance to moving forward.
Intervention adherence	If the adherence rate was ≥84% of the intervention (≥5 out of the 6 coaching sessions) If the rate was between 84–67% (≥5.4 out of the 6 coaching sessions), this needs further discussion to explore the reasons and whether, if they can be modified, then the trial may progress with cautions. If the adherence rate was less than 67%, there is no significance to moving forward.

## Data Availability

All data generated or analysed during this study will be included and are available upon reasonable request from the corresponding author.

## Supplementary Materials

The following supporting information can be downloaded at: https://www.mdpi.com/article/10.3390/ijerph192215089/s1, File S1: CONSORT 2010 checklist of information to include when reporting a pilot or feasibility trial, File S2: BCTs matching the motivational interviewing, partly adopted from Hardcastle et al. (2017) [40], File S3: BCTs matching the stage of change model, partly adapted from Moore et al. (2015) [41].
