# Supplementary material for: Assessing the Feasibility and Acceptability of Health Coaching as a New Diabetes Management Approach for the People with Type 2 Diabetes in Saudi Arabia: A Protocol for a Mixed Methods Feasibility Study
Source: Int J Environ Res Public Health. 2022 Nov 16;19(22):15089. doi: 10.3390/ijerph192215089 (PMC9690217; doi:10.3390/ijerph192215089)
Supplement: Supplementary file 1 [file ijerph-19-15089-s001.zip › ijerph-1981294-supplementary.pdf]

Supplementary File S1

CONSORT 2010 checklist of information to include when reporting a pilot or feasibility trial[31]

Section/Topic	Item No	Checklist item	Reported on page No
**Title and abstract**			
	1a	Identification as a pilot or feasibility randomised trial in the title	1
	1b	Structured summary of pilot trial design, methods, results, and conclusions (for specific guidance see CONSORT abstract extension for pilot trials)	1
**Introduction**			
Background and objectives	2a	Scientific background and explanation of rationale for future definitive trial, and reasons for randomised pilot trial	2–4
	2b	Specific objectives or research questions for pilot trial	4
**Methods**			
Trial design	3a	Description of pilot trial design (such as parallel, factorial) including allocation ratio	4
	3b	Important changes to methods after pilot trial commencement (such as eligibility criteria), with reasons	NA
Participants	4a	Eligibility criteria for participants	5
	4b	Settings and locations where the data were collected	5
	4c	How participants were identified and consented	5
Interventions	5	The interventions for each group with sufficient details to allow replication, including how and when they were actually administered	5–7
Outcomes	6a	Completely defined prespecified assessments or measurements to address each pilot trial objective specified in 2b, including how and when they were assessed	18

	6b	Any changes to pilot trial assessments or measurements after the pilot trial commenced, with reasons	NA
	6c	If applicable, prespecified criteria used to judge whether, or how, to proceed with future definitive trial	19–20
Sample size	7a	Rationale for numbers in the pilot trial	5
	7b	When applicable, explanation of any interim analyses and stopping guidelines	19–20
Randomisation:			
Sequence generation	8a	Method used to generate the random allocation sequence	5
	8b	Type of randomisation(s); details of any restriction (such as blocking and block size)	5
Allocation concealment mechanism	9	Mechanism used to implement the random allocation sequence (such as sequentially numbered containers), describing any steps taken to conceal the sequence until interventions were assigned	5
Implementation	10	Who generated the random allocation sequence, who enrolled participants, and who assigned participants to interventions	5
Blinding	11a	If done, who was blinded after assignment to interventions (for example, participants, care providers, those assessing outcomes) and how	4
	11b	If relevant, description of the similarity of interventions	NA
Statistical methods	12	Methods used to address each pilot trial objective whether qualitative or quantitative	20–21
**Results**			
Participant flow (a diagram is strongly recommended)	13a	For each group, the numbers of participants who were approached and/or assessed for eligibility, randomly assigned, received intended treatment, and were assessed for each objective	NA
	13b	For each group, losses and exclusions after randomisation, together with reasons	NA
Recruitment	14a	Dates defining the periods of recruitment and follow-up	NA
	14b	Why the pilot trial ended or was stopped	NA
Baseline data	15	A table showing baseline demographic and clinical characteristics for each group	NA
Numbers analysed	16	For each objective, number of participants (denominator) included in each analysis. If relevant, these numbers should be by randomised group	NA

Outcomes and estimation	17	For each objective, results including expressions of uncertainty (such as 95% confidence interval) for any estimates. If relevant, these results should be by randomised group	NA
Ancillary analyses	18	Results of any other analyses performed that could be used to inform the future definitive trial	NA
Harms	19	All important harms or unintended effects in each group (for specific guidance see CONSORT for harms)	NA
	19a	If relevant, other important unintended consequences	NA
**Discussion**			
Limitations	20	Pilot trial limitations, addressing sources of potential bias and remaining uncertainty about feasibility	NA
Generalisability	21	Generalisability (applicability) of pilot trial methods and findings to future definitive trial and other studies	NA
Interpretation	22	Interpretation consistent with pilot trial objectives and findings, balancing potential benefits and harms, and considering other relevant evidence	NA
	22a	Implications for progression from pilot to future definitive trial, including any proposed amendments	NA
**Other information**			
Registration	23	Registration number for pilot trial and name of trial registry	NA
Protocol	24	Where the pilot trial protocol can be accessed, if available	NA
Funding	25	Sources of funding and other support (such as supply of drugs), role of funders	NA
	26	Ethical approval or approval by research review committee, confirmed with reference number	4

Supplementary File S2

Table 10. BCTs matching the motivational interviewing, partly adopted from Hardcastle et al., (2017) [41]

MI Technique	Example of Technique	BCTs from Michie et al.'s (2013) taxonomy
ENGAGING TECHNIQUES:		
Affirmation	Despite the fact that things didn't go as you had hoped, your intentions were good."	15.1: Verbal persuasion about capability
FOCUSING TECHNIQUES:		
Elicit-Provide Elicit	"What do you know about type II diabetes? "	5.1: Information about health consequences
EVOKING TECHNIQUES:		
Running Head Start	"What advantages might a change in lifestyle bring?"	9.2: Pros and cons
Looking Forward	"What may occur if the current situation is maintained?"	9.3: Comparative imagining of future outcomes
Hypothetical thinking	"What steps would you take if you did decide to alter your behaviour?"	15.2: Mental rehearsal of successful performance
Query Extremes	"What do you think would be the BEST outcome from this change?"	5.5: Salience of consequences
Identify Past Successes	"What have you discovered after trying to change in the past?"	15.3: Focus on past success
Identify Strengths	"Tell me about your key strengths?"	13.4: Valued self-identity

Troubleshooting	"Imagine supposing there wasn't this one significant roadblock. How might you go about implementing this change if that barrier were removed?"	1.2: Problem-solving
Values Exploration (open or structured)	"What issues are most crucial to you? or "What is your greatest life goal?" How do your eating habits align with your beliefs and goals?"	13.4: Valued self-identity
Reframing	Change "I'm unable to do it" to "So, you find it challenging to…"	13.2: Framing/reframing
Normalizing	"Most people have both positive and negative effects from altering their [target behaviour]" or "Many people report experiencing similar emotions to yours. They try to lose weight, but they have trouble.	6.2: Social comparison
PLANNING TECHNIQUES:		
Explore Change Expectations	"What would you anticipate happening if you choose that course of action?"	1.3: Goal setting (outcome)
Consider Change Options	"What are the things you could do?"	1.4: Action planning
Develop a Change Plan	What do you intend to do specifically?	1.4: Action planning
Goal attainment Scaling	Rate a weight loss goal on a scale ranging from −3 (most unfavourable outcome): gain 5 kg in one month to +3 (most favourable outcome): lose 5 kg in one month where 0 is the status quo (remain at current weight)	1.5: Review behaviour goal(s)
Support Change/Persistence	"How may I help you?"	3.1: Social support (unspecified)
Offer Emotional support	"I can assume that I would feel the same way if I were going through what you are."	3.3: Social support (emotional)
Review Outcome goal	"How far along are you with your objective?"	1.7: Review outcome goal(s)
Summarise the Plan	"So, you are going to do …"	1.9: Commitment

Supplementary File S3

Table 11. BCTs matching the *stage of change model*, partly adapted from Moore et al., (2015) [42]

Stage	Health coaching Skills to use	Matched with BCTs
Pre-contemplation	Empathy	3.3: Social support (emotional)
	Sharing	3.1: Social support (unspecified)
	Acceptance	13.4: Valued self-identity
	Affirming	15.1: Verbal persuasion about capability
	Reframing	13.2: Framing/reframing
	Sorting barriers	1.2: Problem-solving
Contemplation	Affirming	15.1: Verbal persuasion about capability
	Sharing	3.1: Social support (unspecified)
	Brainstorming/Sorting barriers	1.2: Problem-solving
	Motivating	10.2: Social reward<br>3.1: Social support (unspecified)
	Self-Efficacy and Self-Esteem:	
	Verbal persuasion	15.1: Verbal persuasion about capability<br>15:4 Self-talk
	Vicarious experience	6.2: Social comparison
Preparation	Committing	1.9: Commitment
	Identifying steps	1.1 Goal setting (behaviour)
	Explore change expectations	1.3: Goal setting (outcome)

	Identifying potential obstacles	1.2: Problem-solving
	Values Exploration	13.4: Valued self-identity
Action	Having a plan	1.4: Action planning
	Reframing	13.2: Framing/reframing
	Tracking progress	1.7: Review outcome goal(s)
	Normalising	6.2: Social comparison
	New network	3.1: Social support (unspecified)
	Preparing for lapses/facing obstacles	1.2: Problem-solving
Maintenance	Maintaining the network	3.1: Social support (unspecified)
	Valuing achievements	9.3: Comparative imagining of future outcomes
	Self-identity	13.1: Identification of self as a role model
